# Sample size calculation in randomised phase II selection trials using a margin of practical equivalence

**DOI:** 10.1186/s13063-020-04248-8

**Published:** 2020-03-30

**Authors:** Hakim-Moulay Dehbi, Allan Hackshaw

**Affiliations:** 1 Comprehensive Clinical Trials Unit at University College London (UCL), 90 High Holborn, London, WC1V 6LJ UK; 2 Cancer Research UK & UCL Cancer Trials Centre, 90 Tottenham Court Road, London, W1T 4TJ UK

**Keywords:** Selection design, Randomised trial, Rare cancers

## Abstract

**Background:**

In rare cancers or subtypes of common cancers, a comparison of multiple promising treatments may be required. The selected treatment can then be assessed against the standard of care (if it exists) or used as a backbone for combinations with new, possibly targeted, agents. There could be different experimental therapies or different doses of the same therapy, and either can be done in combination with standard treatments. A ’pick-the-winner’ design is often used, which focuses on efficacy to select the most promising treatment. However, a treatment with a slightly lower efficacy compared to another treatment may actually be preferred if it has a better toxicity or quality of life profile, is easier to administer, or cheaper.

**Methods:**

By pre-defining a margin of practical equivalence in order to calculate the sample size, a more flexible assessment can be made of whether the treatments have very different effects or are sufficiently close so that other factors can be used to choose between them. Using exact binomial probabilities, we calculated the sample size for two- and three-arm randomised selection trials including a margin of practical equivalence with a variety of input parameters.

**Results:**

We explain conceptually the margin of practical equivalence in this paper, and provide a free user-friendly web application to calculate the required sample size for a variety of input parameters.

**Conclusion:**

The web application should help promote the randomised selection design with a margin of practical equivalence, which provides greater flexibility than the ’pick-the-winner’ approach in assessing the results of selection trials.

## Background

Rare cancers, defined as those with an incidence of less than 6 cases per 100,000 individuals per year, together account for 20–25% of all cancers in Europe and the USA [1, 2]. Furthermore, given the increasing use of genomic-based classification of tumours and the identification of tumour markers for which licenced or experimental targeted therapies are available, more rare molecularly defined subgroups of common cancers are expected [3]. Conducting large-scale phase III trials in rare tumours or subgroups is often not feasible, and therapies are instead investigated using phase II studies.

Many phase II trials (single-arm or randomised) are designed for superiority of the experimental therapy against a current standard of care or occasionally placebo, in which a certain efficacy threshold needs to be met in order for the therapy to warrant further investigation or be approved for use in clinical practice. There are also multi-arm designs that assess several experimental therapies at the same time, i.e. different agents, different doses of the same agent, or a combination of these. Multi-arm phase II selection studies often use a ’pick-the-winner’ decision rule, in which the chosen or preferred treatment is simply the one with the highest efficacy numerically [4]. A recent example is the InterAACT trial [5], which is the first prospective randomised study of first-line treatment for patients with inoperable locally recurrent or metastatic squamous cell carcinoma of the anus. The main goal is to compare the doublet combination of cisplatin and 5-fluorouracil with carboplatin and paclitaxel, to determine which would become the optimal chemotherapy backbone to combine with experimental targeted agents in future studies. Because the pick-the-winner approach focusses on efficacy only, the decision rule in InterAACT was considered alongside toxicity and quality of life (QoL). If the trial groups had equal tumour response rates (TRR), the treatment with the fewest severe toxicities would be chosen, and if both efficacy and toxicity were equal between the groups, then QoL would be used to make the choice [6]. This more comprehensive strategy for choosing the ‘best’ therapy is appealing, and forms the motivation for our paper.

A problem associated with using endpoints other than efficacy to choose between treatments, is first determining ‘equal/similar’ efficacy, because two treatments will rarely have identical results for an outcome measure. Sargent and Goldberg attempted this by developing a trial design which incorporates a pre-defined margin that allows investigators to choose a treatment that is slightly less efficacious than another, but instead has better toxicity or QoL, is easier to administer, or cheaper [7]. This ‘margin of practical equivalence’ would be agreed on by the trial investigators, and would represent the extent by which a treatment needs to be superior so that it is selected based on efficacy only.

Sargent and Goldberg described the design for a two-arm trial and provided a few sample size tables based on a limited set of possible input parameters. Their design has been cited more than 50 times. However, it is rarely used in practice because the publication was fairly statistical, making it difficult to understand for the medical audience, and the design is not included in any sample size calculation software.

The aims of our paper are to promote the adoption of the randomised selection design using a margin of practical equivalence by explaining it in an intuitive manner, extend the design to three arms, and provide a user-friendly web application to allow investigators to calculate the required sample size.

## Methods

### The two-arm selection trial with a margin of practical equivalence

#### Decision rule

**Fig. 1:**
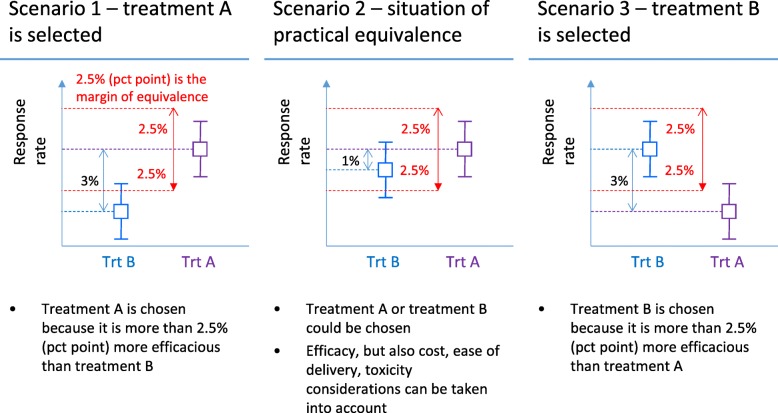
Decision rule of the randomised selection trial design with a margin of practical equivalence under the three possible scenarios

Figure 1 presents the decision rule of the randomised selection design with a margin of practical equivalence, which we explain here using a numerical example.

Let us assume that two treatments, drug A and drug B, are compared, and the measure of efficacy is the TRR. The true underlying TRRs of A and B are assumed to be 20% and 10% respectively, with a true underlying difference of 10 percentage points (we call this *delta*). We define a margin of practical equivalence, called *d,* and here we fix this at 2.5 percentage points. These four quantities (the true TRRs of drugs A and B, *delta*, and *d*) are referred to as input parameters throughout this paper.

The decision rule at the end of the trial comprises three scenarios (see Fig. 1):

1. If the observed TRR of drug A compared to drug B is higher by more than *d*, which is 2.5 percentage points, drug A is selected (e.g. a TRR of 22% with drug A and 15% with drug B), based on efficacy only, and regardless of toxicity or other factors.
2. If the observed TRR of drug A is below that of drug B, but the difference is less than 2.5 percentage points, this represents practical equivalence (e.g. a TRR of 15% with drug A and 16% with drug B). This situation can be reversed, in that drug B could have the lower TRR. In either of these two cases, we then consider other factors such as toxicity to choose between drugs A and B.
3. If the observed TRR of drug A is below that of drug B, and the difference is at least 2.5 percentage points, then drug B would be chosen, regardless of toxicity or other factors.

#### Sample size

To calculate the sample size, we use the probability of selecting the truly most efficacious treatment, called *P*_most_. To calculate how many patients are needed for each trial arm, we make sure that *P*_most_ is above a certain threshold, which we can set at ≥ 80% (and is analogous to power).

The most efficacious treatment is selected in two circumstances at the end of the trial:

1. If the most efficacious treatment has an observed TRR that is more than *d* percentage points greater than that of the other treatment (the probability of correctly selecting that treatment in this circumstance is *P*_correct_)
2. If we choose the most efficacious treatment in situations of practical equivalence (the probability of a situation of equivalence is denoted *P*_equi_). Assuming that the other considerations of interest (e.g. toxicity, cost, QoL) are unrelated to the TRR, the most efficacious treatment is selected 50% of the time in situations of practical equivalence, as we only have two options.

Mathematically, this means that *P*_most_ is equal to *P*_correct_ + 50% * *P*_equi_.

We refer to the possibility that the least efficacious treatment is chosen at the end of the study as *P*_wrong_. This takes place when by chance the least efficacious treatment has an observed TRR that is more than *d* percentage points greater than that of the most efficacious treatment. Overall, *P*_correct_ + *P*_equi_ + *P*_wrong_ = 1.

Exact binomial probabilities, or Monte Carlo simulations, can be used to derive the sample size required so that *P*_most_ is above a pre-specified threshold (see Additional file 1). In Table 1, we provide sample sizes for *P*_most_ of 80% or 85%, a margin of practical equivalence *d* of either 2.5 or 5 percentage points, a *delta* of 10 percentage points, and different TRRs for the two drugs.

**Table 1:** Sample size per arm necessary for a two-arm trial, for a fixed *delta* of 10 percentage points, a margin *d* of 2.5 or 5 percentage points, and *P*_most_ at 80% or 85%. The tumour response rate with drug A varies between 20 and 80%

		Margin *d* 2.5%		Margin *d* 5%	
TRR drug A	TRR drug B	*P*_most_ 80%	*P*_most_ 85%	*P*_most_ 80%	*P*_most_ 85%
20%	10%	19	28	19	35
30%	20%	27	46	32	54
40%	30%	33	53	37	59
50%	40%	36	57	39	73
60%	50%	36	57	39	73
70%	60%	33	53	37	70
80%	70%	27	46	32	54

The selection of the most efficacious treatment takes place under two circumstances: (1) when its observed TRR is higher than that for any other treatment(s) by more than the margin (*P*_correct_), or (2) if it is selected when practical equivalence occurs (*P*_equi_). Consequently, the size of *P*_correct_ within *P*_most_ is an important consideration in the sample size calculation. For similar levels of *P*_most_, increasing the sample size increases the share of *P*_correct_ and reduces the share of *P*_equi_. It might be reasonable to decide on a sample size with a minimum threshold for *P*_correct_, and this must be agreed on by the study team, including the clinician(s).

For situations of practical equivalence, the assumption that the other considerations of interest (e.g. toxicity, cost, QoL) are unrelated to the TRR may or may not be valid. In general terms, the probability of selecting the most efficacious treatment can be specified as *P*_correct_ + ρ * *P*_equi_ = *P*_most_. Using ρ = 0 means that the most efficacious treatment is never selected in situations of practical equivalence. This implies that *P*_most_ = *P*_correct_ and the required sample size is larger than if one assumes ρ = 50%. Although using ρ = 0 is likely to be unrealistic in practice, it can be used to calculate an upper range of the sample size. Similarly, using ρ = 1 can be used to calculate a lower range of the sample size.

### Extending to three-arm designs

Designing a three-arm trial follows the same principles as in a two-arm trial. As in the two-arm setting, we are interested in ensuring that the most efficacious treatment is correctly selected. The sample size required should be such that *P*_most_ exceeds a certain high threshold (e.g. 80%).

With three arms, *P*_correct_ corresponds to the case where the observed TRR of the most efficacious drug is *d* percentage points greater than the TRRs of the other two treatments. For *P*_equi_, a situation of practical equivalence occurs if:

1. One of the two pairwise comparisons is practically equivalent (defined as in the two-arm setting), or
2. The two pairwise comparisons are practically equivalent.

Case 1 corresponds to a situation where one of the two less efficacious treatments is in a situation of practical equivalence with the most efficacious treatment, while the other less efficacious treatment is outside the margin. Case 2 corresponds to a situation where all three treatments are within the margin of practical equivalence.

In case 1, the most efficacious treatment is selected 50% of the time, as we are choosing between two options. This assumes that the other considerations of interest (e.g. toxicity, cost, QoL) are unrelated to the TRR. In case 2, the most efficacious treatment is selected 33% (a third) of the time, because we are choosing between the three treatments, if we make the same assumption of unrelatedness of efficacy with the other considerations.

In mathematical terms, *P*_most_ is equal to *P*_correct_ + 50% * *P*_equi (case 1)_ + 33% * *P*_equi (case 2)_.

Exact binomial probabilities or Monte Carlo simulations can be used to establish the required sample size for three-arm selection trials, for any input parameters.

As in the two-arm setting, the chance of selecting the most efficacious treatment in situations of equivalence may not be 50% (in case 1 of equivalence) and 33% (in case 2 of equivalence). These percentages assume that the other considerations of interest are unrelated to the TRR. Replacing these probabilities by 0% provides an upper range of the sample size that is solely based on *P*_correct_. Similarly, if we replace these probabilities by 100%, we obtain the lower range of the sample size that assumes that in all situations of practical equivalence the most efficacious treatment is selected.

## Results

### A free user-friendly web application

**Fig. 2:**
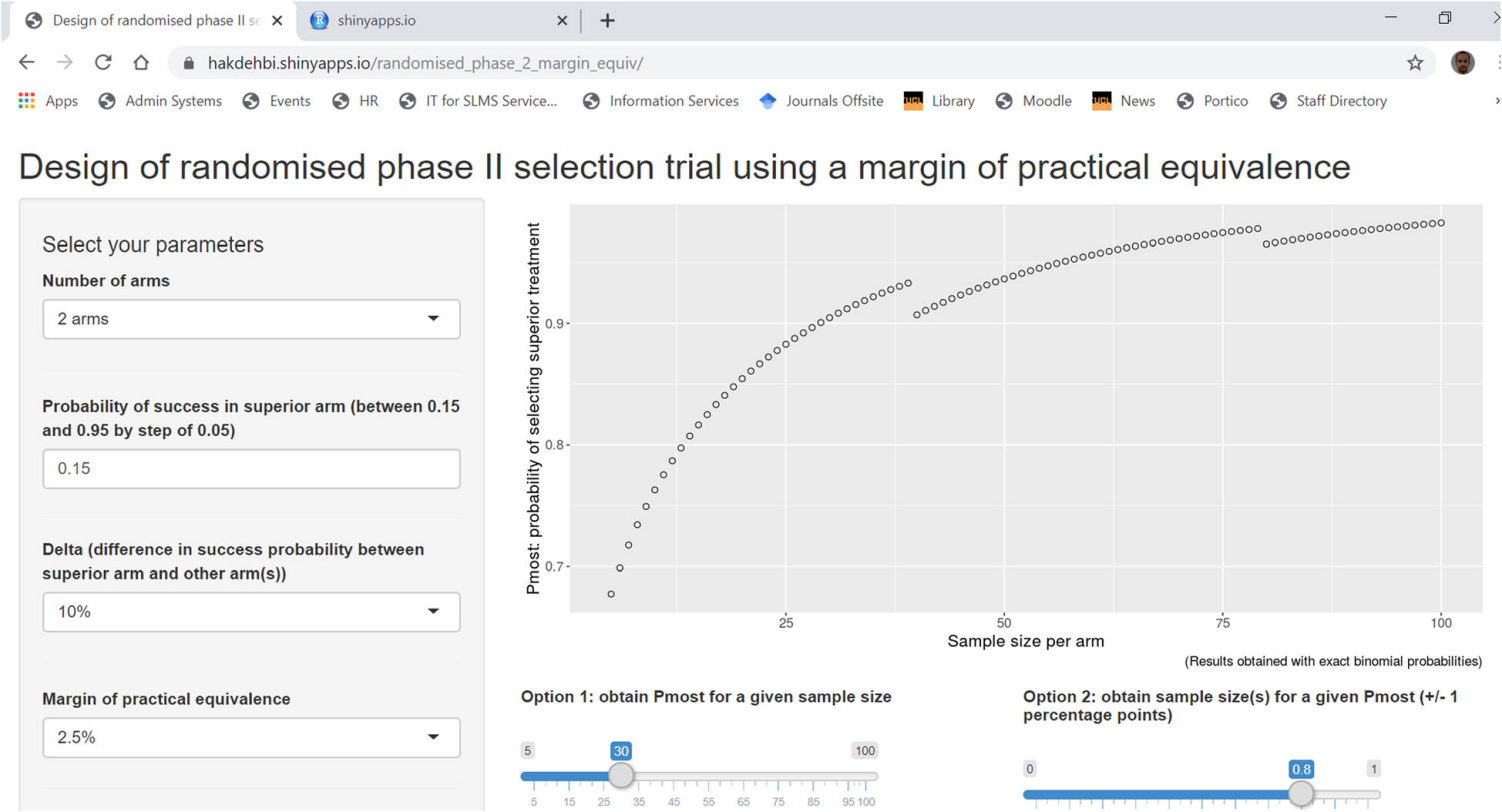
Online application to calculate the sample size for a randomised two- or three-arm selection trial with a margin of practical equivalence

To facilitate the use of the randomised selection design with a margin of practical equivalence, we developed a free online application—see Fig. 2. It can be found at https://hakdehbi.shinyapps.io/randomised_phase_2_margin_equiv/. The application provides the user with a comprehensive set of possible combinations of input parameters: the margin *d* of practical equivalence can be chosen as 2.5 or 5 percentage points (which should be appropriate for most situations), the *delta* as 10 or 15 percentage points, and the TRR in the superior arm varies between 15 and 95% in steps of 5%. The output allows the calculation of the required sample size for a specific *P*_most_ or to derive *P*_most_ for a given sample size. The software provides sample sizes that were calculated using exact binomial probabilities.

Figure 2 shows that a wide range of sample sizes provide similar values of *P*_right_ (plotted on the vertical axis). For example, in a two-arm trial in which the superior treatment has a TRR of 15% with a *delta* and *d* of 10 and 5 percentage points respectively, 35 patients per arm would provide 89% for *P*_most_, while 54 patients would provide 91%. For similar levels of *P*_most_, increasing the sample size tends to increase the share of *P*_correct_ within *P*_most_ . It is therefore possible to design a selection trial with a minimum level for *P*_correct_ as well as a target value for *P*_most_.

## Discussion

The International Rare Cancers Initiative (IRCI) was set up in 2011 and comprises organisations involved in trials in rare cancers from several countries, including the UK, USA, Canada, and France [8]. One of the IRCI’s objectives is to promote the development of innovative methodologies for research in rare cancers. This paper contributes to this objective for multi-stage trials where the first stage consists of choosing from two or more promising treatments. Formally incorporating a margin of practical equivalence in the design, and calculating the sample size accordingly, allows researchers to determine when the choice of therapy should be made only on the efficacy outcome measure, or when it can be done on the basis of other factors (e.g. toxicity) because efficacies are considered similar. This design provides a more flexible and realistic approach when deciding which treatment among several should be investigated further.

In the randomised selection design of Simon et al. [4], the sample size is calculated so that there is a high probability of correctly selecting a superior treatment for further testing in phase III, if such a treatment exists. However, if the treatments truly have equal efficacy, the treatment with the highest observed response rate will be declared superior, even though this is not true. In other words, there is no control of the false positive rate. Another practical limitation is that the situation where the observed response rates are equal is not considered, although this can happen, especially with small sample sizes and binary endpoints. Even when using a margin of practical equivalence, the minimum difference in number of patients between arms may be just one patient. For example, this would be the case if 19 patients are used per arm and a margin of 5 percentage points is used. Indeed one patient out of 19 represents 5.2%. Nonetheless, the advantage of the proposed approach is that it allows researchers to formalize, at the start of the study, how the extra considerations (cost, QoL, etc.) might be used in the decision rule if the treatments have equal response rates.

To overcome these limitations, recent selection trials have included in their protocols rules based on toxicity, QoL, and survival to choose a treatment when the observed response rates are equal/similar. Examples of such ongoing trials are InterAACT [5], COSMIC [9], and NEOSCOPE [10]. In the ongoing InterAACT trial, toxicity and QoL will be used to make the selection if the response rates are equal. However, if response rates are very similar but not equal, the treatment with the highest observed rate will be selected even if the difference might be due to chance. In the COSMIC trial of chemotherapy plus ofatumumab at standard or high dose in chronic lymphocytic leukaemia, the protocol stipulates that ’if there are less than 3 responses (8%) difference observed between the arms, the trial will be declared statistically ambiguous, and alternative selection criteria will be used to select the schedule for further investigation. Otherwise, the schedule with the better observed response rate will be recommended to be taken forward’. Interestingly, the additional criteria to make the selection (if less than three responses difference are observed between arms) are not specified. In NEOSCOPE, a randomised trial of induction chemotherapy followed by either oxaliplatin/capecitabine- or paclitaxel/carboplatin-based chemoradiation as a pre-operative regimen for resectable oesophageal cancer, the protocol includes specific rules based on survival and toxicity to make the selection if efficacy is comparable between arms. Effectively, these three trials employ a margin of practical equivalence in their decision rule. However, the sample size calculations were performed without taking into account the margin, potentially leading to reduced power compared to the planned target study size. In InterAACT and COSMIC it was assumed that one of the two treatments had a higher efficacy than the other, while in NEOSCOPE the sample size was calculated as if the two arms were independent single-arm phase II studies.

Sample size calculations based on traditional phase III trial designs often lead to unfeasibly large sample sizes in rare cancers. Recruitment is a major challenge, given the low incidence and the geographical spread of patients with rare cancers across countries. In InterAACT, 388 patients and 25 years of recruitment would be required to demonstrate an increase in TRR from 40 to 50% using carboplatin and paclitaxel compared to cisplatin with 5-fluorouracil, with the traditional superiority design and 80% power at a 5% two-sided significance level [6]. With the design of Simon et al., 36 patients were needed per arm to demonstrate the same increase from 40 to 50%, which has an 80% chance of selecting the superior treatment at the end of trial (the researchers increased this number to 40 per arm for logistical reasons). If a margin of practical equivalence of 5 percentage points had been used, 38 patients per arm would have been required, but with the advantage of having a more flexible and pre-planned strategy for choosing the ‘best’ treatment, if treatments demonstrate equal or similar efficacy in the study. In general, the sample size required for a selection trial is larger when a margin is incorporated, compared to designs that do not include a margin; and this difference in study size gets larger as *d* increases. Keeping all other input parameters fixed, the smallest sample size is reached when the margin *d* is fixed at zero (corresponding to the design of Simon et al.). In other words, there is a trade-off between sample size and the flexibility introduced by the margin.

In practice, for a given sample size, if the true difference in TRR between treatments is not as large as was expected in the sample size calculation, the chance to select the most efficacious treatment is reduced. For example, 19 patients are required per arm if we use a TRR of 20% for the most efficacious treatment, a *delta* of 10 percentage points (i.e. the TRR of the other treatment is expected to be 10%), a *P*_most_ of 80% and a margin of 5 percentage points; see Table 1. If the true difference is less than 10 percentage points, the chance of selecting the most efficacious treatment (which happens if there is more than one patient difference between the two arms) is reduced to less than 80%. If both treatments have a TRR of 20%, both treatments would be chosen based on efficacy only 42% of time. In 16% of the time, the study would be in a situation of practical equivalence. Assuming that both treatments have the same or comparable profiles on the non-efficacy considerations, then the study has 100% chance to make an appropriate choice, as both treatments are equal. However, if one treatment is noticeably better in terms of toxicity and/or QoL, then it would be chosen only slightly more than 50% of the time. Indeed it would be chosen based on efficacy only 42% of the time, and at least 50% of the time in situations of practical equivalence (16% chance of such a situation happening). This example demonstrates the importance of determining the *delta* realistically, as well as the importance of setting the margin *d* in such a way that making a decision based on efficacy considerations only is acceptable medically.

Nonetheless, it is possible that a treatment is considered superior based on efficacy considerations only at the end of the study, but only by a very small margin. In such circumstances, the non-efficacy considerations may still be taken into account in the final decision, especially if crucial non-efficacy considerations emerged during the trial. The decision rule might then be seen as a guideline or starting point for making a judgment on which treatment should be taken for further testing. Additionally, the determination of the margin *d* may take into account potential prior information on non-efficacy considerations. In general, the design assumes that the treatments are equal with respect to non-efficacy considerations, prior to starting the study. However, if this is not the case, the margin may be made wider at the calculation stage to reflect the additional gain in efficacy that the treatment eventually taken further should demonstrate to compensate for the slightly reduced QoL or increased toxicity, for example.

We note that the sample size depends on the absolute values of the TRRs, which should be determined using the best available evidence to date. For the same *delta* of 10 percentage points in Table 1, the sample size increases when the middle point of the [0,1]-axis for the TRR is approached compared to the boundaries of the [0,1]-axis. This is due to the bounded nature of the binomial distribution.

The proposed design is based on binary outcome measures; hence, a limitation is that it does not allow for other types, i.e. continuous or time-to-event endpoints. However, binary outcomes can be used for either short (e.g. TRR as in our example) or longer term measures (e.g. 1- or 2-year progression-free or overall survival). Another potential advantage of the proposed design is for use in biomarker-directed early phase treatment trials, in which arms could be compared with a control therapy.

The user-friendly online application should help promote the randomised selection design with a margin of practical equivalence. Users currently have the choice between two- or three-arm trials, a margin *d* of 2.5% or 5%, and a *delta* of either 10% or 15%. There might be other options in the future, but in the meantime users can contact us if they wish to specify other values for the input parameters that are not currently available within the web application.

Although our paper was developed primarily for treatment trials for uncommon cancers, the methods can be applied to other cancers too, particularly when a smaller efficacy study is considered appropriate or more feasible as an initial assessment of a therapy. Moreover, this method may be applicable for randomisation purposes at the end of a phase I dose-finding trial when there remains considerable uncertainty in the selection of the dose to take for further testing.

## Supplementary information

**Additional file 1.** Sample size calculation in randomised phase II selection trials using a margin of practical equivalence.

## Data Availability

Not applicable.
