# Supplementary material for: Sample size calculation in randomised phase II selection trials using a margin of practical equivalence
Source: Trials. 2020 Mar 30;21:301. doi: 10.1186/s13063-020-04248-8 (PMC7106856; doi:10.1186/s13063-020-04248-8)
Supplement: Supplementary file 1 — Additional file 1. Sample size calculation in randomised phase II selection trials using a margin of practical equivalence. [file 13063_2020_4248_MOESM1_ESM.pdf]

COMPREHENSIVE CLINICAL TRIALS UNIT AT UCL

Sample size calculation in randomised phase II selection trials using a margin of practical equivalence

Hakim-Moulay Dehbi, PhD

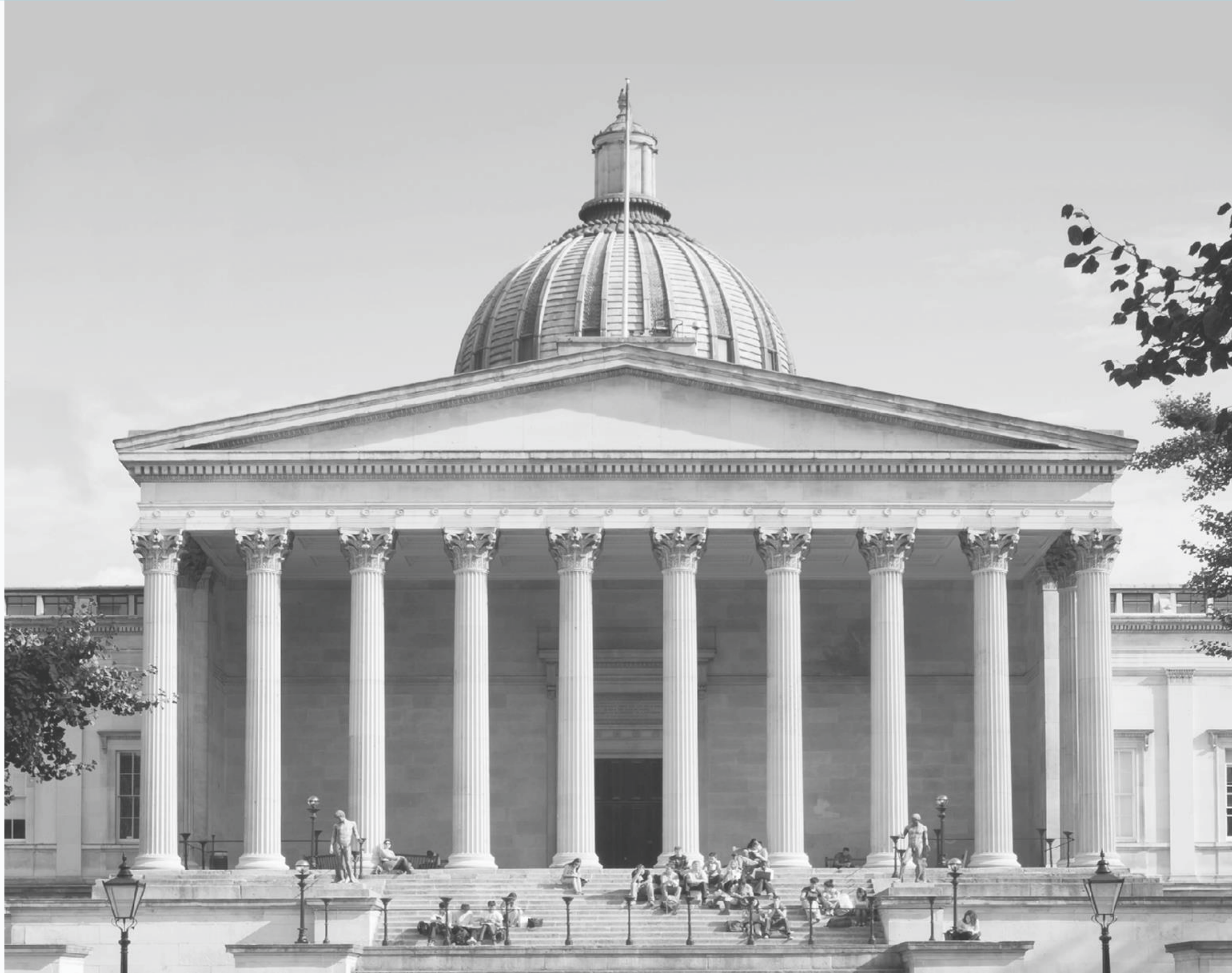

Statistical design of selection trial with margin of equivalence

- Two treatments:
  - Treatment A: π_a true response rate, P_a observed rate
  - Treatment B: π_b true response rate, P_b observed rate
- Goal: ensure that if one treatment is clearly superior, there is a large probability that it is selected
- Define d as margin of practical equivalence, and assume Trt A is clearly superior with $\pi_a > \pi_b + \delta$
  - δ is true difference in response rate between treatments (δ > d)
- 3 probabilities of interest for the 3 possible situations at end of study
  - $P_{cor} = \Pr[P_a > P_b + d \mid \pi_a, \pi_b]$
  - $P_{equiv} = \Pr[P_b - d \leq P_a \leq P_b + d \mid \pi_a, \pi_b]$
  - $P_{wrong} = \Pr[P_b > P_a + d \mid \pi_a, \pi_b]$

- Define $\lambda = P_{cor} + 50\% * P_{equiv}$
  - λ is the probability is correctly choosing the superior treatment
  - 50% * P_{equiv} as one chance out of two of choosing A in case of equivalence (assuming efficacy of A and B is unrelated to the other factors of interest (toxicity, QoL, etc…))
- Using exact binomial probabilities, determine N (sample size per arm) so that $\lambda > \gamma$
  - generally $\gamma \geq 80\%$

How to calculate P_{cor}, the proba that Trt A is indeed chosen as superior when it is the case

- 20 patients per arm
- π_a = 50%
- π_b = 40%
- Margin: 5%
  - ≥ 2 patients difference between A and B to correctly select A
- #A is number of response with Trt A
  - #A ~ Bin (20, 0.5)
- #B is number of response with Trt B
  - #B ~ Bin (20, 0.4)

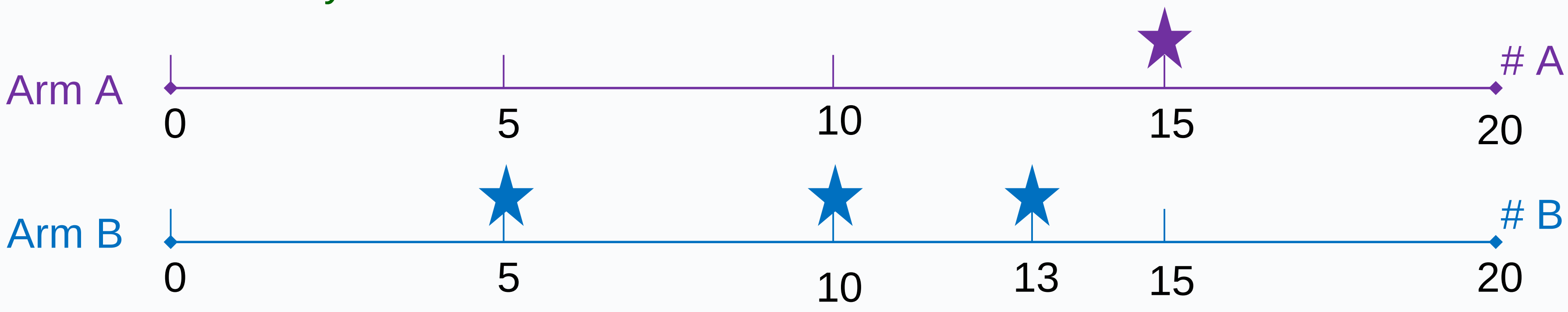

- $P_{cor} = \sum P(\# A \mid \pi_a) * P(\# B \mid \pi_b)$
  - Summation over all combinations of #A and #B such that (#A - #B) ≥ 2

How to calculate P_{equiv}, the proba that Trt A is indeed chosen as superior when it is the case

- 20 patients per arm
- π_a = 50%
- π_b = 40%
- Margin: 5%
  - -1, 0, 1 patient difference between A and B implies a situation of equivalence
- #A is number of response with Trt A
  - #A ~ Bin (20, 0.5)
- #B is number of response with Trt B
  - #B ~ Bin (20, 0.4)

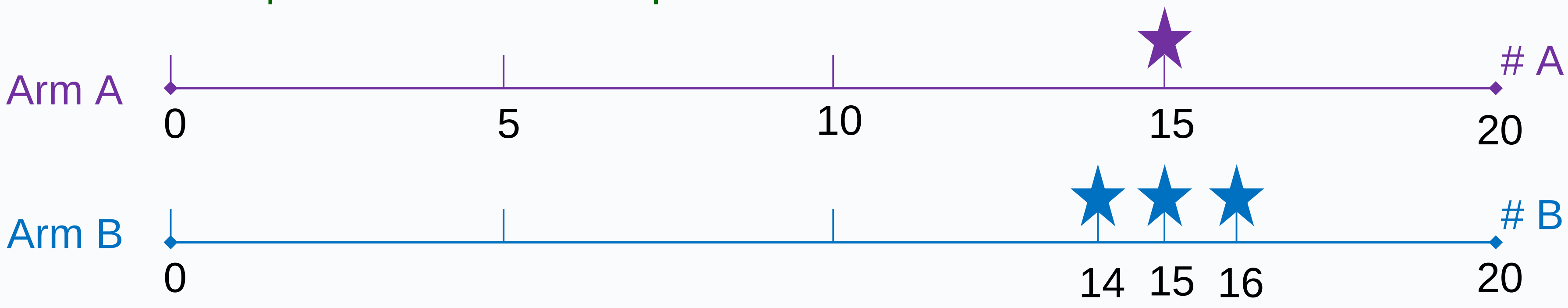

- $P_{equiv} = \sum P(\# A \mid \pi_a) * P(\# B \mid \pi_b)$
  - Summation over all combinations of #A and #B such that -1 ≤ (#A - #B) ≤ 1

How to calculate P_{wrong}, the proba that Trt b is wrongly chosen as superior

- 20 patients per arm
- π_a = 50%
- π_b = 40%
- Margin: 5%
  - ≤ -2 patient difference between A and B corresponds to a situation where B is chosen
- #A is number of response with Trt A
  - #A ~ Bin (20, 0.5)
- #B is number of response with Trt B
  - #B ~ Bin (20, 0.4)

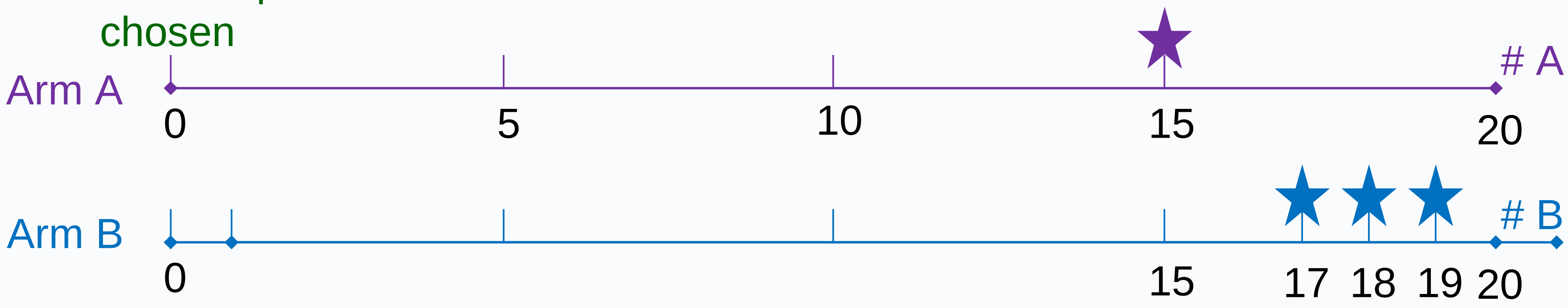

- $P_{wrong} = \sum P(\# A \mid \pi_a) * P(\# B \mid \pi_b)$
  - Summation over all combinations of #A and #B such that (#A - #B) ≤ -2

Sample size calculator

https://hakdehbi.shinyapps.io/randomised_phase_2_margin_equiv/

Contact details

Hakim-Moulay Dehbi, PhD

Comprehensive Clinical Trials Unit at UCL

Institute of Clinical Trials & Methodology

90 High Holborn, 2nd floor, London WC1V 6LJ

Email: h.dehbi@ucl.ac.uk
